# Research on point cloud hole filling and 3D reconstruction in reflective area

**DOI:** 10.1038/s41598-023-45648-5

**Published:** 2023-10-28

**Authors:** Chao Sun, LongXin Miao, MeiYuan Wang, Jiuye Shi, JianJun Ding

**Affiliations:** 1 State Key Laboratory of Precision Blasting, Jianghan University, Wuhan, 430056 China; 2 College of Intelligent Manufacturing, Jianghan University, Wuhan, 430056 China; 3 College of Artificial Intelligence and Automation, Huazhong University of Science and Technology, Wuhan, 430074 China

**Keywords:** Engineering, Mathematics and computing, Optics and photonics, Physics

## Abstract

3D reconstruction is the process of obtaining the three-dimensional shape or surface structure of an object, which is widely used in advanced manufacturing fields such as automotive, aerospace, industrial inspection, and reverse engineering. However, due to the structural characteristics of the component itself, the reflective properties of the coating material, and other factors, there may be specular reflection during image acquisition, making it difficult to achieve complete 3D reconstruction of the component. This paper proposes a method to address the problem of incomplete 3D reconstruction of strongly reflective objects by recognizing outlier points and filling point cloud holes. The proposed View-Transform-PointNet outlier point recognition network improves the alignment of the initial point cloud plane and implements secondary alignment of the point cloud based on the perpendicularity between the outlier plane in mixed reflection and the point cloud plane. The point cloud hole-filling method is based on the principle of outlier formation and approximates a local Gaussian distribution to linear variation. The distance between the end of each outlier plane and the real surface is calculated to repair the depth information of outlier points. The proposed method achieves a 39.4% increase in the number of point cloud filling, a 45.2% increase in the number of triangular mesh faces, a 46.9% increase in surface area, and a chamfer distance (CD) of 0.4471009, which is better than existing geometric repair methods in terms of standard deviation and smoothness. The method improves the alignment of initial point cloud planes and enhances the accuracy of outlier point recognition, which are the main innovative points of this study. The 3D reconstruction of the repaired point cloud model is achieved through Poisson equation and parameter adjustment. The proposed method reduces the error caused by large curvature in the boundary region and improves the smoothness and accuracy of the reconstructed model.

## Introduction

**Figure 1:**
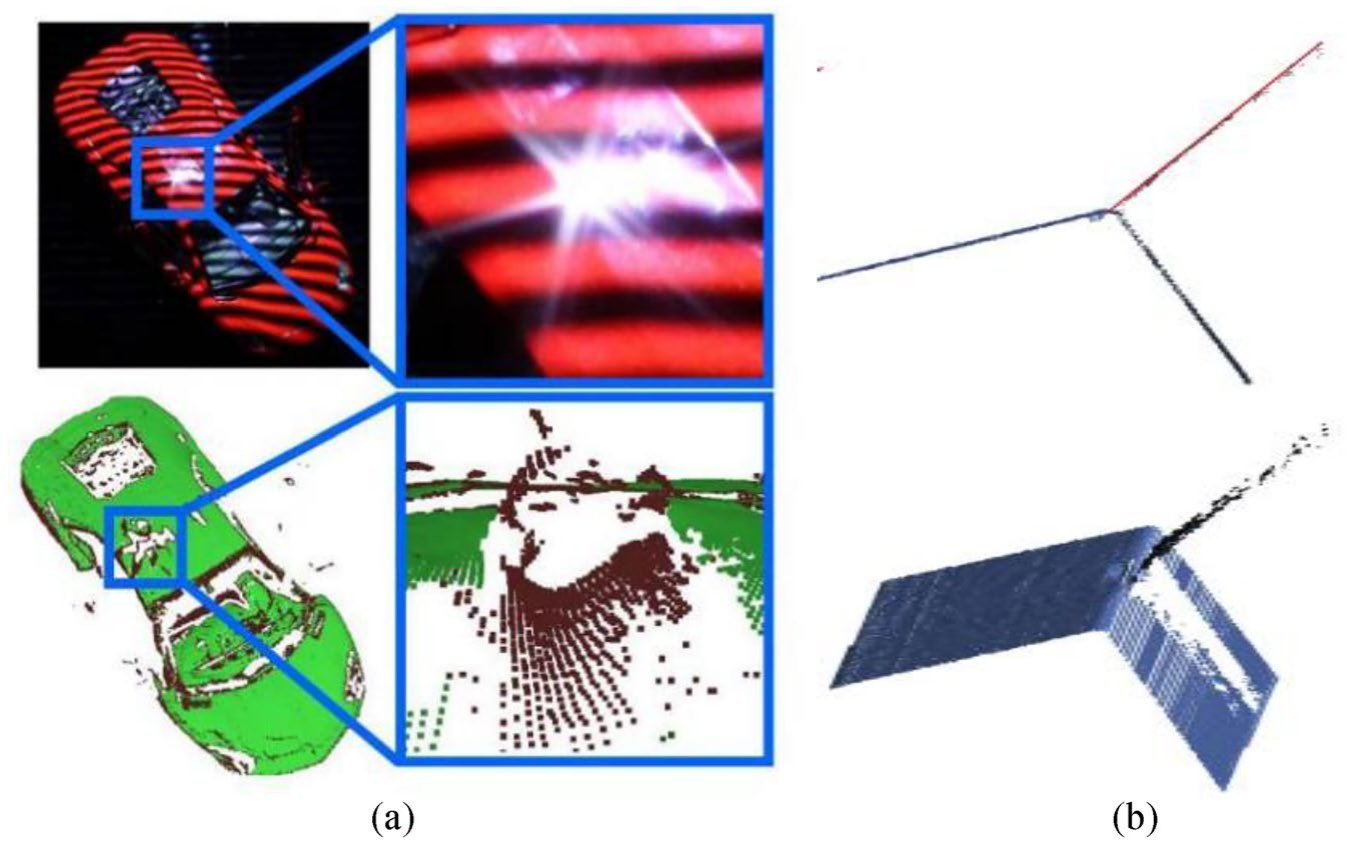
Schematic diagram of outliers during 3D scanning. (**a**) When scanning strong reflective areas^5^, (**b**) when scanning object edge features^6^.

3D reconstruction technology is the process of acquiring the shape or surface structure of an object^1^. With the rapid development of 3D scanning and computer technology, 3D point cloud reconstruction technology has been widely applied in various fields such as computer vision, 3D mapping, and robotics^2–4^.The existing optical 3D scanning methods are mainly two methods: laser 3D scanning systems and structured light scanning systems. Among them, the structured light 3D scanning system, using Laser triangulation to obtain 3D coordinate information of the object surface, has the advantages of simple system structure, fast scanning speed, and high point cloud resolution. However, due to the surface nature of the object under test, the light source, and the complex structure, the structured light 3D scanning system inevitably suffers from noise pollution when scanning the surface of the object, producing the corresponding outlier noise (as shown by Fig. 1), it results the absence of point cloud information in some areas of the scanned object, forming point cloud holes, which greatly impairs the quality of the image captured by the imaging device.

For outliers generated in the reflective region, they are typically regarded as noise points and are removed in subsequent point cloud processing. Alternatively, high-reflectance surface outliers can be eliminated by analyzing images captured by cameras^7,8^. However, outliers generated in the reflective region during structured light scanning are related to the surface characteristics of the scanned object^6^. Unlike other studies that use information around point cloud voids for repair through methods such as curve fitting and interpolation, the repair of these outliers can be achieved by utilizing their location information in the point cloud.

In this paper, we propose a method based on View-Transform perspective transformation for the 3D reconstruction of strongly reflective components, and improve the alignment network in PointNet to achieve automatic identification and extraction of outlier points generated from highly reflective surfaces; in addition, a point cloud hole repair method based on outlier points is proposed for the point cloud hole problem generated by outlier point clouds; Finally, through Poisson surface reconstruction of the repaired point cloud, the 3D surface reconstruction of the highly reflective components is realized, and the shortcomings of the hole repair method are effectively compensated, which provides a new research idea for the subsequent 3D reconstruction of highly reflective objects.

## Related work

### Noise removal and 3D reconstruction of reflective surfaces

A projector is used to discriminate a small number of outlier points based on point cloud consistency^6^. To handle these outlier points, improvements were made to the hardware equipment by using dual cameras to fuse the scanned reflective areas and then reconstruct the point cloud. This method effectively solves the problem of outliers generated by structured light scanning on reflective surfaces. In addition, a projector is used to discriminate a small number of outlier points based on point cloud consistency^5^. A non-local self-similarity-based point cloud denoising method can preserve sharp feature information using adaptive curvature thresholding, propose a projection height vector perceptron, and introduce a weighted parametric optimization height matrix to achieve high-precision reconstruction^9^.The MaskNet++ framework for inner-outer point detection uses spatial self-attention and channel cross-attention mechanisms to identify outlier noise in point clouds, effectively removing isolated points and outlier points^10^. The proposed type-based outlier removal framework quantifies the input point cloud features by three parameters and divides the point cloud to reject outliers by the proposed single-criterion method^11^. This method effectively solves the problem of outliers generated by structured light scanning on reflective surfaces.

### Filling point cloud holes

The research primarily focuses on analyzing the point cloud information around the hole. By analyzing the features of the point cloud around the hole, the point cloud around the hole is transferred to the point cloud hole area, generating point cloud information similar to the surrounding features. After refinement and adjustment, the point cloud can be smoothly transitioned^12^. A mesh deformation-based approach to digitized hole filling is proposed that relies on a priori knowledge of the numerical model as a nominal mesh and performs the deformation of the nominal mesh after identifying the digitized holes and calculating the difference between the nominal mesh and the point cloud, which is effectively verified on industrial parts^13^.A method to repair point cloud holes by combining FPFH with ICP algorithm for registering and fusing the processed point cloud model with the original point cloud^14^. The method can also perform local repair by dividing the point cloud holes into small patches based on feature lines, and then using NURBS to fit surfaces to gradually achieve repair of the patches and complete the global repair^15^. Similarly, it is difficult to guarantee the quality of the repair results after improving the object of the motion structure and structured light and fusing multiple sensor point sets for alignment to complete the hole repair^16^.

### Point cloud repair based on deep learning

Pu-Net repair network can generate denser and more homogeneous point clouds from sparse point cloud sets, but cannot repair large holes or missing parts^17^. Introducing 2D convolutional networks into 3D point clouds generates multiple projection views from different perspectives, and uses CNN networks to repair at the image level, but the projection angle causes missing point cloud information in the image^18^. From the perspective of voxelization, the point cloud is sampled at different resolutions to generate voxel grids, and point cloud repair is achieved through voxel-based deep learning networks. However, this approach requires a large number of samples, leading to long training times and high computational costs^19^. The PF-Net network uses a multi-scale repair network based on feature points to predict missing point clouds^20^.

### Point cloud 3D reconstruction

Efficient and accurate algorithms have been proposed successively according to the differences in the expressions and principles of reconstructed surfaces. In the work of surface reconstruction using parameters, there are methods of B-sample surfaces, non-uniform rational B-sample surfaces, and triangular Bezier surfaces^21–23^, which have the advantages of being computationally simple, easy to display, and having geometric invariance. However, the parameterization requirements are high to ensure that the reconstructed surfaces can accurately reflect the shape and characteristics of the original data. Based on non-quadratic sampling discrete shear transform and Laplace algorithm to calculate the focus degree of image sequence pixels, and complete the 3D reconstruction of milling tool tip based on the out-of-focus shape principle^24^. The point clouds are aligned by RGB-D depth images in different time frames, thus enabling digital 3D reconstruction of the vehicle and endowing it with texture information^25^. In the reconstruction of 3D surfaces using neural networks, Sign Agnostic Learning learns implicit representations from point clouds with triangular meshes to achieve surface reconstruction of undirected point clouds^26^; deep learning networks are introduced to integrate the surface and learn to modify the indicator function parameters from undirected noisy point clouds to generate smooth surfaces with high normal consistency^27^. This restoration method has high approximation capability and high reconstruction accuracy for point cloud surfaces, but it is difficult to converge and has high resource consumption.

## Methodology

### PointNet outlier recognition network based on view transform

**Figure 2:**
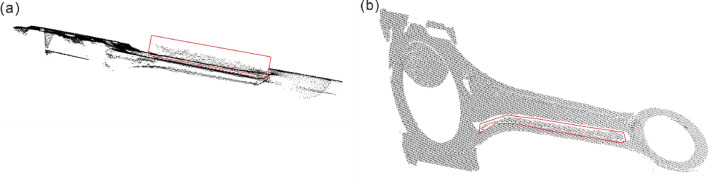
Outliers formed by optical reflection (**a**) Outliers formed by optical reflection due to concave structure; (**b**) Outliers are arranged in an orderly manner from a specific perspective.

During the 3D point cloud measurement of highly reflective components using direct projection laser triangulation method, there are outliers in some specific structures, such as concave structures and regions with large curvature changes, due to optical reflections. As shown in Fig. 2a, the point cloud inside the red box exhibits outliers. However, they exhibit a certain pattern when viewed from a specific angle, as shown in Fig. 2b. The following section will explain the principle behind this phenomenon.

**Figure 3:**
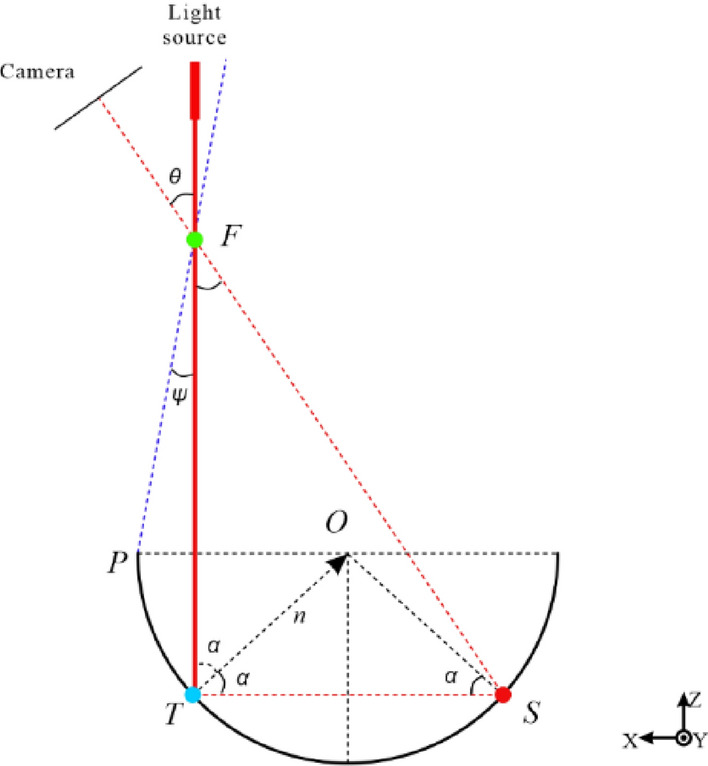
Principle of Multipath Reflection.

The reason for this is due to the multipath reflection that occurs when scanning concave geometric structures. The principle of multipath reflection is shown in Fig. 3.

In the Fig. 3, when scanning a concave geometric model using structured light, the structured light source illuminates the concave surface, and the camera captures diffuse reflection light from the true surface information point $T$. However, due to the existence of the concave surface structure, a specular reflection occurs at point $T$ and generates an erroneous surface information point $S$ inside the concave structure through secondary reflection. The camera also captures the diffuse reflection light generated by the secondary reflection and records it as an outlier point $F$. That is, the camera captures two images of diffuse reflection generated during one scan: one from the true surface information $T$ and one from the erroneous surface information $S$ generated by the secondary reflection, and records the outlier point $F$. The relationships between the various parameters are as follows:

1 $$ \psi = tan^{ - 1} \left( {\frac{{\left( {1 - \sin \alpha } \right)\sin \theta }}{{2\cos \alpha \sin \left( {2\alpha + \theta } \right) - \cos \alpha \sin \alpha }}} \right) $$

In the equation, α represents the angle between the structured light source and the normal vector n, θ represents the angle between the camera and the structured light source, and ψ represents the angle between the structured light source and the edge of the concave structure.

**Figure 4:**
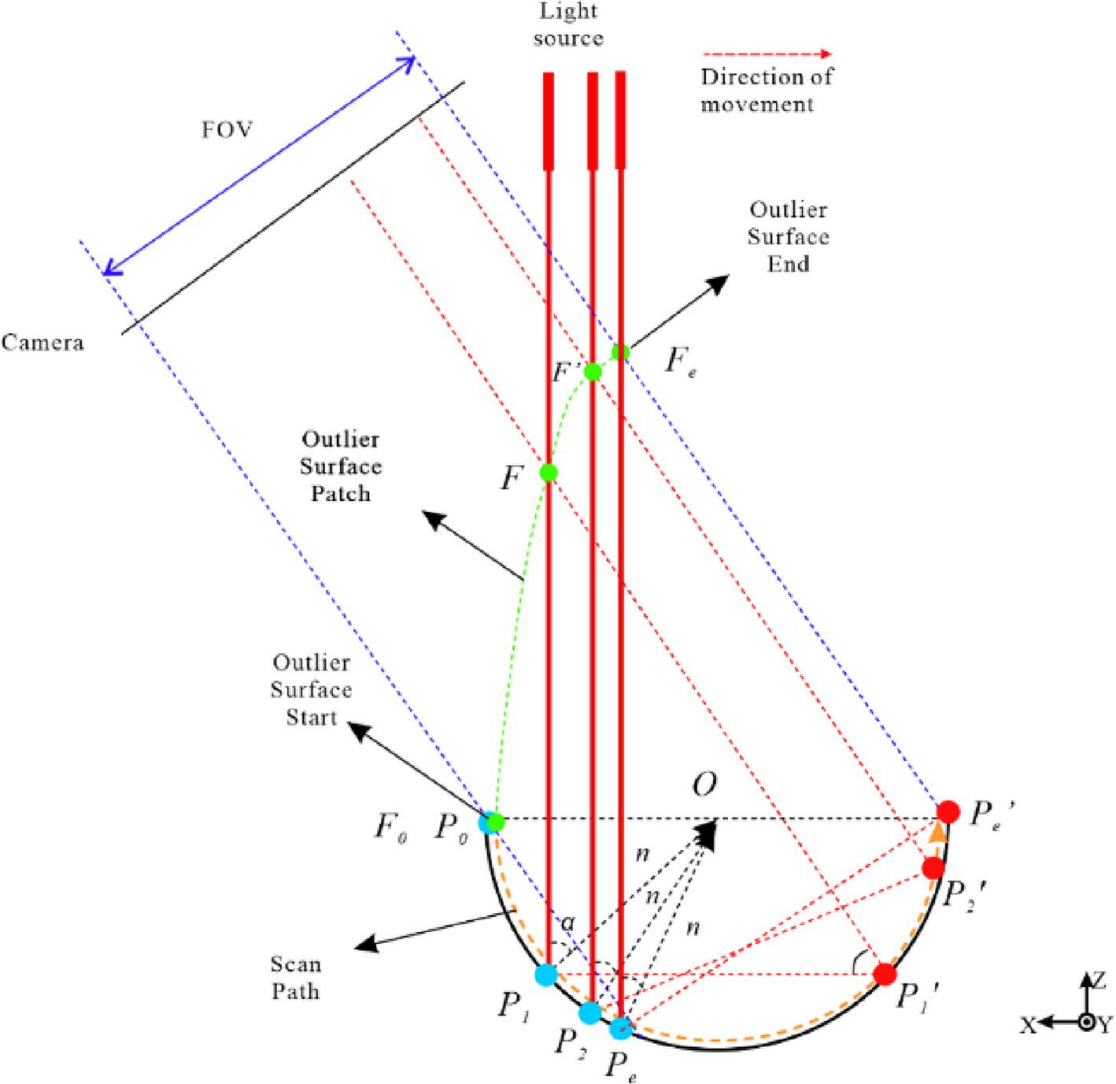
Multipath reflection during scanning process.

When the structured light source gradually scans the concave surface structure, the formation of its outliers is shown in the following Fig. 4:

The orange dashed line in the Fig. 4 represents the scanning path, the blue dashed line represents the field of view of the concave structure on both sides in the camera, the red solid and dashed lines represent the reflection paths of the scanned light, and the green dashed line represents the path generated by the outlier plane. The blue dots represent the real points $P_{1}$, $P_{2}$, $P_{e}$ generated on the concave structure during the structured light scanning path. The secondary reflection formed on the right side of the concave structure generates diffuse reflection points $P_{1}^{\prime }$, $P_{2}^{{^{\prime } }}$, $P_{e}^{{^{\prime } }}$ and the outliers generated by the secondary reflection are $F$, $F^{{^{\prime } }}$, and $F_{e}$, respectively.

Without considering the field of view (FOV) of the camera, the formation of outliers can be explained as follows: as the structured light scans the surface from left to right, it first contacts the edge of the concave surface at the starting point $P_{0}$, which is also the starting point of the outlier plane formation path. As the structured light scans further, real points $P_{1}$ and $P_{2}$ are present on the left side of the concave surface and secondary reflections are formed within the concave surface, resulting in the formation of $P_{1}^{{^{\prime } }}$ and $P_{2}^{{^{\prime } }}$, which are captured by the camera as outliers $F$ and $F^{{^{\prime } }}$. At the same time, the angle $\alpha$ between the structured light source and the normal vector $n$ decreases continuously. However, as α decreases and the structured light scans to $P_{e}$, the secondary reflection is located at the edge of the concave surface, forming diffuse reflection $P_{e}^{{^{\prime } }}$, and the camera captures the last secondary reflection to form outlier $F_{e}$, which marks the end of the outlier plane formation path. In the subsequent scanning path, since the secondary reflection lacks a supporting surface, multiple reflections no longer occur and the outliers disappear.

However, in practical scanning, the camera field of view cannot be ignored, as shown by the blue dashed line in the Fig. 4, and the camera can only capture images within the camera field of view. During scanning, the image of the structure light source on the left within the dashed line cannot be captured by the camera due to occlusion caused by the concave surface structure. Only the image generated by the secondary reflection within the camera field of view can be captured. Therefore, in reality, this area should be a hole region, but due to the presence of the secondary reflection, a point cloud can still be formed through the gray centroid method. However, the point cloud in this area is an outlier.

The point cloud outliers formed by multipath reflections exhibit the following characteristics: 1. The outliers form multiple outlier planes; 2. The outlier planes exhibit two-dimensional features; 3. The outliers exhibit ordered characteristics in two specific viewpoints, but appear disordered in other viewpoints. Therefore, feature extraction for point cloud outliers cannot ignore the viewpoint, i.e., the adjustment of the point cloud pose.

**Figure 5:**
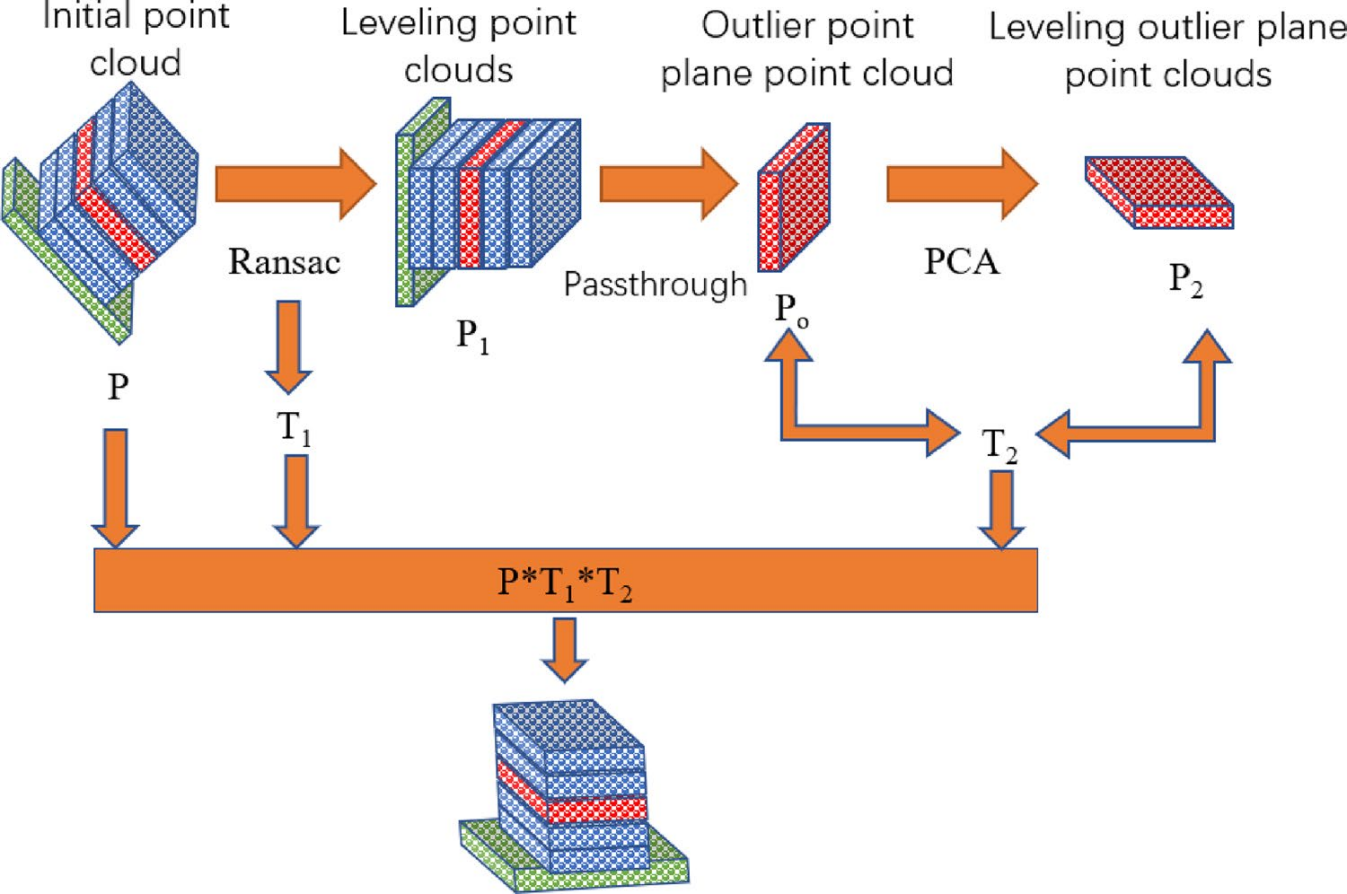
View-Transform method.

Therefore, this article proposes a View-Transform point cloud rotation method, which rotates the initial point cloud to an ordered perspective based on the characteristics of point cloud outliers, thereby improving the feature extraction of outliers in deep learning networks and improving the recognition accuracy of point cloud outlier recognition networks. The process is shown in Fig. 5:

1. Initial point cloud leveling: Identify the middle plane of the initial point cloud *P* through the random sampling consensus method, where the middle plane of the point cloud is the platform plane, then level the point cloud through principal component analysis (PCA), and solve the registration and leveling point cloud and the initial point cloud through singular value decomposition (SVD) to obtain the preliminary rotation matrix $T_{1}$;
2. Point cloud direct filtering: In order to avoid the influence of other point clouds, obtains two-dimensional outlier plane point clouds $P_{o}$ with obvious features through direct filtering. The formula is:
  2 $$ \left\{ {\begin{array}{*{20}c} {P_{o} \left( {x,y,z} \right) x \in \left[ {x_{1} ,x_{2} } \right] y \in \left[ {y_{1} ,y_{2} } \right] z \in \left[ {z_{1} ,z_{2} } \right]} \\ \\ {P_{o} \left( {x,y,z} \right) \in P\left( {x,y,z} \right)} \\ \end{array} } \right. $$
  $x_{1} ,x_{2} ,y_{1} ,y_{2} ,z_{1} ,z_{2}$ are respectively the ranges of the *XYZ* axes for the pass-through filter used to filter the initial point cloud $P$, and $P_{o}$ is the resulting outlier plane point cloud.
3. The normal vector of the outlier plane is solved through PCA, and the point cloud is rotated in the direction of $n$ to obtain the outlier point plane point cloud $P_{2}$. The formula for solving the normal vector is as follows:
  3 $$ \mathop  \to \limits_{{n}}  = \left\{ {\begin{array}{*{20}c}    {M_{{min}} ~~~~~~~~~M = \frac{1}{k}\sum\nolimits_{{i = 1}}^{k} {\left( {p_{i}  - p_{0} } \right)\left( {p_{i}  - p_{0} } \right)^{T} } }  \\    \left\Vert{\mathop  \to \limits_{n}}\right\Vert  = 1  \\    {\mathop  \to \limits_{{n_{i} }} *\mathop  \to \limits_{{n_{j} }}  \approx 1}  \\   \end{array} } \right. $$
  Among them, *M* is the sample variance, find the minimum value*.* $p_{0}$ is the centroid of the point cloud, where $\to _{{n_{i} }} *\to _{{n_{i} }} \approx 1$ is the ambiguity of the restricted normal vector. The positive and negative directions of the point cloud normal vector are determined by multiplying the adjacent two points normal vectors.
4. Point cloud registration adjustment, achieved by Iterative Closest Point(ICP) for outlier plane point cloud $P_{o}$ and outlier plane point cloud $P_{2}$, get the rotation matrix $T_{2}$;
5. To adjust the initial point cloud pose, multiply the initial point cloud *P* with the rotation matrices $T_{1}$ and $T_{2}$, obtaining the point cloud $P^{\prime}$ adjusted to the optimal viewing angle, with the specific transformation formula:
  4 $$ P^{\prime} = P*T_{1} *T_{2} $$

The initial point cloud is rotated to the viewpoint where the outlier features are most prominent using the above method, in order to achieve better identification accuracy in subsequent outlier detection networks for point clouds.

### Hole repair of structural light point cloud of strong reflective component

When light is reflected on the surface of an opaque object, there are generally three different types of reflection components: diffuse reflection lobes, specular reflection lobes, and specular spike reflection lobes. When the camera is located in the specular reflection lobe or the specular spike reflection lobe, overexposure will occur, leading to the generation of outliers. In this case, the surface normal vector will be on the angle bisector of the angle between the light source and the camera, which is called the forbidden normal.

**Figure 6:**
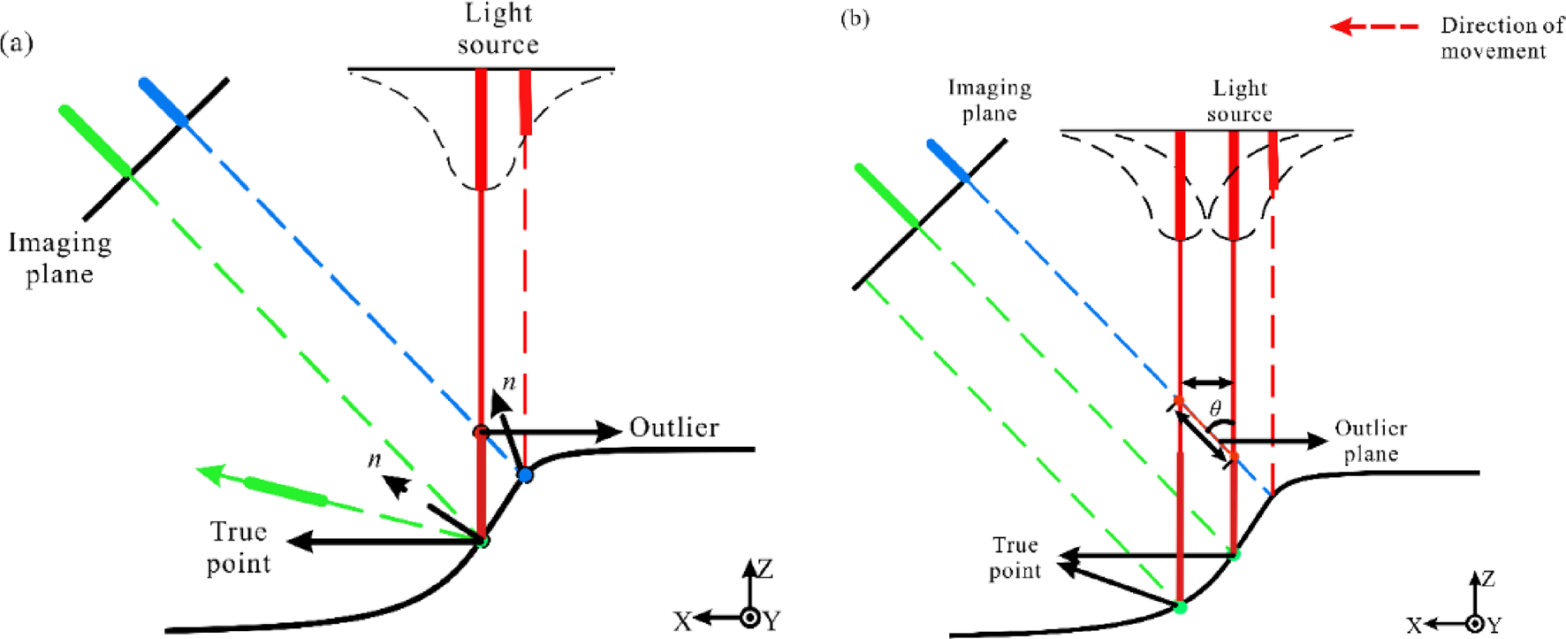
Schematic diagram of outliers and outlier planes in the mixed reflection model (**a**): outliers generated at a certain time; (**b**) Outlier planes generated during continuous scanning.

Due to the influence of mixed reflection, a large number of outlier points are generated when scanning rays are parallel to the concave feature surface of the edge, leading to unsatisfactory reconstruction results of the 3D model. The principle of the outlier points generated by the mixed reflection model at some point in time is shown in Fig. 6.

The solid red line in the Fig. 6 represents the source light, which is the highest intensity center light area (spot area); the solid blue line is the non-central light area; the diffuse reflection light (green) and specular reflection light (green, not captured by the camera) are represented by the corresponding color length indicating the light intensity of each laser. The laser source emits Gaussian laser stripes on the surface, producing diffuse reflection and specular reflection. The diffuse reflection light (green line) follows a Gaussian distribution, but only accounts for a portion of the light intensity of the light source (indicated by the length of the green bar). The CCD sensor on the left captures the diffuse reflection, detects the peak, and records a point on the scanning surface (green dot). Due to the Gaussian distribution of laser intensity, non-central light areas of the laser will be irradiated in the edge area during the entire scanning process, where the surface normal continuously changes. The camera then receives the specular reflection light from this area, as shown by the blue line in Fig. 6a. Although the light intensity of this area is smaller than that of the center light, when this large portion of light intensity is reflected by the specular reflection, the light intensity received by the camera will become higher, forming a local peak. Therefore, the sensor camera has two peak points mixed together, one from the diffuse reflection light of the center light on the side, and the other from the specular reflection light of the edge area with a forbidden normal. When the light intensity is high enough, the peak of specular reflection will also become the recorded data point. Such outlier points (red), current value points (green), and true value points (blue) have a large deviation, resulting in incorrect data points.

When the laser moves to the left, the distance between the diffuse reflection area of the laser center and the specular reflection area of the non-central light gradually increases, and the number of outliers also increases, along with the distance between the outliers and the true scanning plane, forming an outlier plane as shown in Fig. 6b. Due to the weaker light intensity on both sides of the Gaussian distribution of the light source intensity, the intensity of the specular reflection will decrease to the point where it is not captured by the camera, and the density of the outlier plane will become sparse as it moves away from the scanning plane. The relationship between the outlier plane and the displacement of the movement is:

5 $$ d_{o} = \frac{{d_{l} }}{sin\theta } $$

In this equation, $d_{l}$ represents the distance between the current laser scanning plane and the outlier plane, while $d_{o}$ is the distance between each generated outlier point and the starting point of the outlier plane at each time step. θ denotes the angle between the laser scanning plane and the field of view of the CCD camera. From the above relationships, it can be concluded that the angle of the outlier plane is only related to θ.

**Figure 7:**
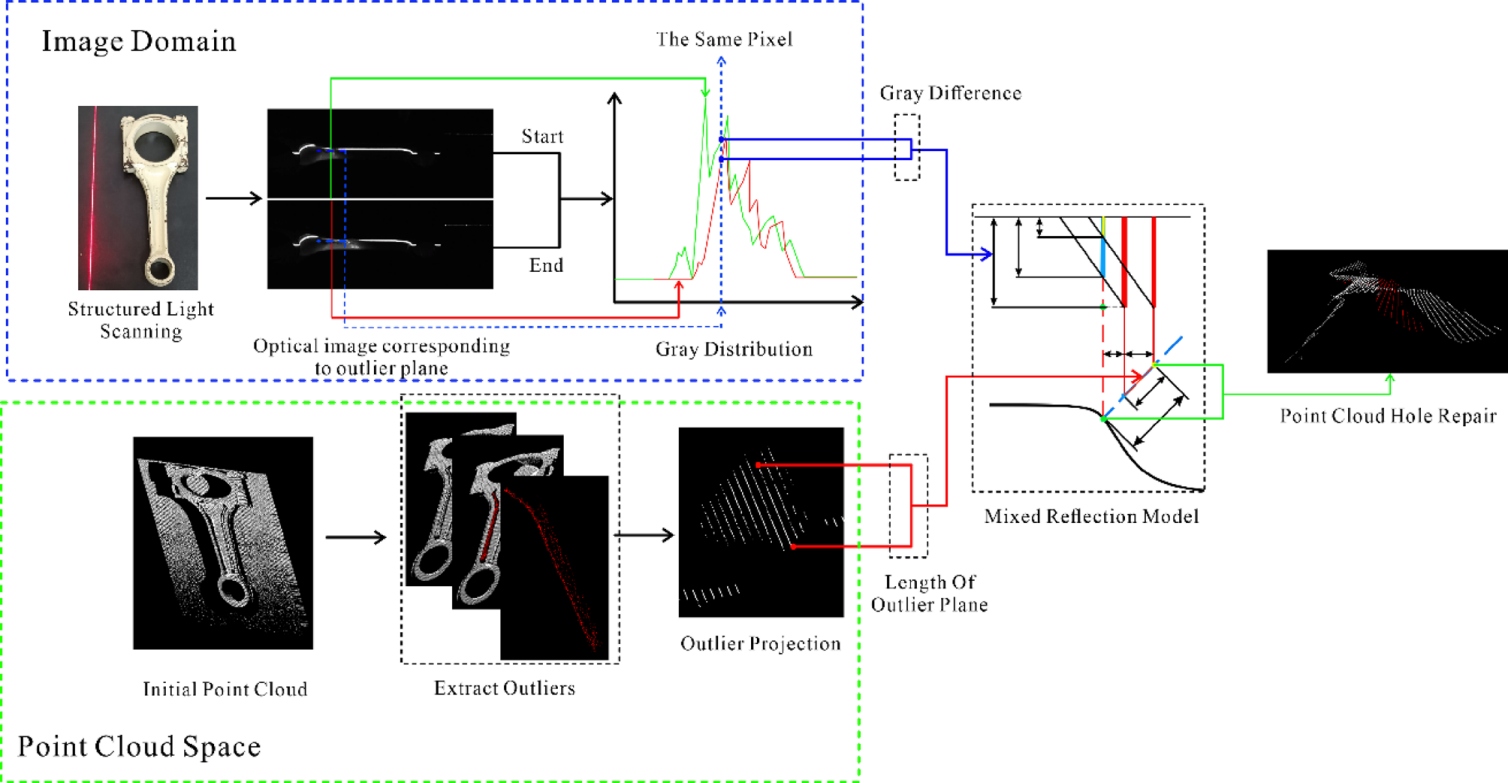
Diagram of the proposed method.

Therefore, accurate repair of outlier position information can effectively solve the problem of outliers and holes in the 3D point cloud model of the measured object. In the paper, based on the analysis of the principle of mixed reflection model, a point cloud hole repair method is proposed based on the principle of mixed reflection model and the characteristics of structural light intensity distribution, and by scanning the WD-2 point cloud model of the automotive connecting rod workpiece, the position information of the outlier is repaired and the filling of the point cloud holes is completed (Fig. 7).

Based on the above analysis of the principle of the mixed reflection model and the structured light cross-sectional grayscale distribution characteristics, it is important to explore the relationship between the location information characteristics of the outlier planes and the grayscale distribution of the corresponding structured light cross-section. Combining the cross-sectional grayscale distribution of the line structured light with the model of mixed reflection, it formed a mapping relationship between the distribution characteristics of the outlier points of the 3D point cloud and the grayscale distribution of the line structured light cross-section, it achieve the restoration of the outlier planar point cloud of the 3D point cloud, as follows:

1. The three-dimensional point cloud information is acquired from the line structured light scanning, which is used to obtain the corresponding two-dimensional point cloud information by projecting the outlier planes to the corresponding planes. As the corresponding equation as:
  6 $$ f_{xoz} \left( {P_{i} \left( {x,y,z} \right)} \right) = P_{i} \left( {x,0,z} \right) $$
  where $f_{{{\text{xoz}}}} \left( \cdot \right)$ is the projection in the $xoz$ plane; $P_{i} \left( {{\text{x}},{\text{y}},{\text{z}}} \right)$ is represented as the point cloud position information.
2. From the existing built triangulated laser measurement equipment, the actual line structure light grayscale distribution, and the structural simplification of the optical principle can be obtained, as shown in Fig. 8.

**Figure 8:**
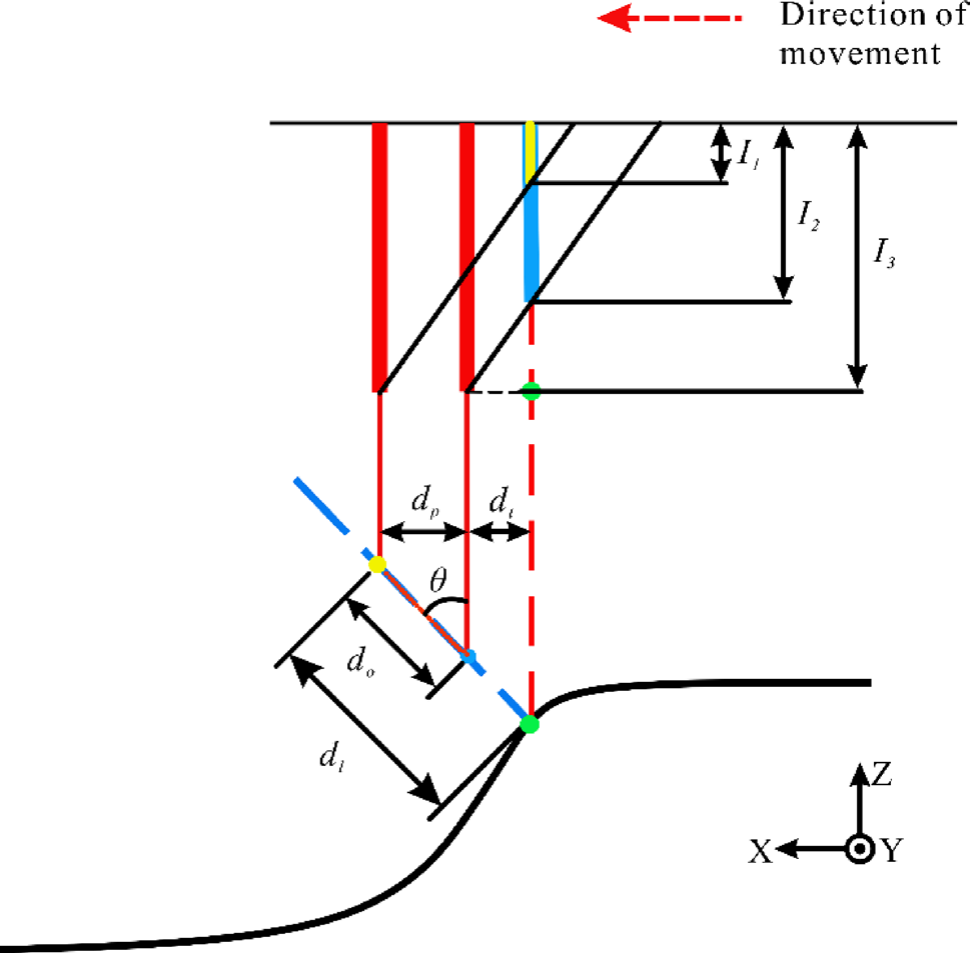
Structure diagram of outlier plane under mixed reflection model.
  where $P_{1} \left( {X_{1} ,Y_{1} ,Z_{1} } \right)$ is the end point of the outlier plane, $P_{2} \left( {X_{2} ,Y_{2} ,Z_{2} } \right)$ is the start point of the outlier plane, thus, the length of the outlier plane can be calculated as:
  7 $$ d_{o} = \sqrt {\left( {X_{1} - X_{2} } \right)^{2} + \left( {Y_{1} - Y_{2} } \right)^{2} + \left( {Z_{1} - Z_{2} } \right)^{2} } $$
  where $$ d_{o}$$ is the length of the outlier plane; since the projection is made in the $$xoz$$ direction, both $$Y_{1} ,Y_{2}$$ are 0.
  The part of the fundamental relations of the optical model in Fig. 7 can be taken as:
  8 $$ \sin \theta = \frac{{d_{p} }}{{d_{o} }} $$
  9 $$ k_{o} = tan\left( {90^\circ - \theta } \right) $$
  where $$\theta$$ is the angle between the field of view of the CCD camera and the structured light; $$d_{p} $$ is the platform displacement distance;$$ k_{o}$$ is the slope of the outlier plane.
3. In practice the characteristics of the laser makes the structured light Gaussian distribution more concentrated, making the actual line structured light grayscale distribution more linear, the line structured light grayscale distribution is considered as a linear variable in this paper.
  Where the line structure light grayscale at the moment of $T_{1}$ and $T_{2}$ corresponds to the line structure light grayscale of $I_{1}$ and $I_{2}$ respectively (obtained from the line structure light grayscale distribution diagram), and the relational equation of the line structure light grayscale distribution is:
  10 $$ k_{l} = \frac{{I_{2} - I_{1} }}{{d_{p} }} $$
  where $I_{1}$ and $I_{2}$ are the line structured light grayscale values; $k_{l}$ is the slope of line structured light grayscale change.
  From Fig. 8, obtain:
  11 $$ sin\theta = \frac{{d_{p} + d_{t} }}{{d_{l} }} $$
  12 $$ d_{t} = \frac{{I_{3} - I_{2} }}{{k_{l} }} $$
  where $d_{t}$ is the distance between the maximum value of line structured light grayness and the corresponding line structured light grayness at the moment of $T_{2}$; $d_{l}$ is the distance between the end of the outlier plane and the real surface.
4. From the above listed expressions, the distance between the end of the current outlier plane and the real surface can be deduced:
  13 $$ d_{l} = d_{o} + k*d_{o} $$
  14 $$ k = \frac{{I_{3} - I_{2} }}{{I_{2} - I_{1} }} $$
  where $k$ is the coefficient of grayscale variation of line structured light.
5. Let the real surface point cloud coordinates be set as $P_{3} \left( {X_{3} ,Y_{3} ,Z_{3} } \right)$, then it would be under this ${ }xoz$ projection plane as:
  15 $$ \left\{ {\begin{array}{*{20}c} { X_{3} = X_{1} + d_{l} *\sin \theta } \\ { Y_{3} = 0 } \\ { Z_{3} = Z_{1} + d_{l} *\cos \theta } \\ \end{array} } \right. $$
6. Therefore, the point cloud information of the real plane generated from the above outlier planes is:
  16 $$ P_{3} = P_{3} \left( {X_{3} ,Y_{1} ,Z_{3} } \right) \cup P_{3} \left( {X_{3} ,Y_{2} ,Z_{3} } \right) \cdots \cup P_{3} \left( {X_{3} ,Y_{n} ,Z_{3} } \right) $$
  where $Y_{1} \sim Y_{n}$ are the Y-axis coordinate values of all the point clouds on this outlier plane.
7. Repeat (1) to (7) above to calculate the distances of all outlier plane ends from the real surface and obtain the corresponding surface point clouds.

Through theoretical analysis and actual measurement, the angle $\theta$ between the field of view of the CCD camera and the structured light is 60°; the length $d_{o}$ of the outlier plane is extracted by the point cloud visualization software; $I_{1}$ and $I_{2}$ can be extracted by the image analysis software for the structured light grayscale distribution of the cross-section; $I_{3}$ is the maximum value of 255 for the line structured light grayscale.

The above algorithm can obtain the missing position information of outlier planes $d_{l}$, and by repairing the outlier information, we can obtain the position information of the actual surface and realize the work of filling the missing parts of the point cloud caused by mixed reflections.

## Experimentation and analysis

### Data collection and preprocessing

**Figure 9:**
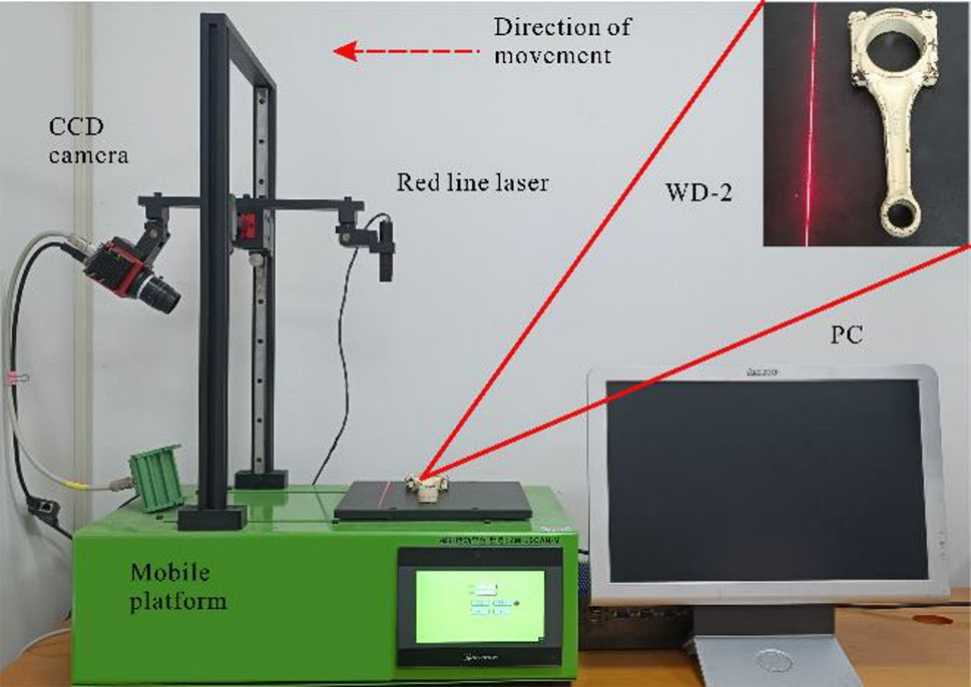
Direct laser triangulation image acquisition system.

The experiments are conducted with the image acquisition system of direct line structured light, including a line infrared laser model FU650AB100-GD16-WLD with a power of 100mW and a wavelength of 650 nm red light. The CCD camera model is C2-2040-GigE with a resolution of 2048 × 1088 pixels. The moving platform model is ZM-LSCAN-M, moving speed is 2.5 cm/s. The detected object used to occur obvious mixed reflections phenomenon is the automobile connecting rod workpiece, the specific model is WD-2. The platform for building a direct laser triangulation image acquisition system is shown in Fig. 9.

The one-line infrared laser is mounted in the vertical direction of the moving platform, and the CCD camera is angled at 60° with the laser to capture the infrared laser lines in the field-of-view area. During the operation of the mobile platform, the CCD continuously acquires the infrared structured light images of the car connecting rod surface. When the image acquisition equipment passes through the mixed reflection area, it will produce extra light bars, which will also be acquired by the CCD and finally generate the 3D point cloud model of WD-2.

**Figure 10:**
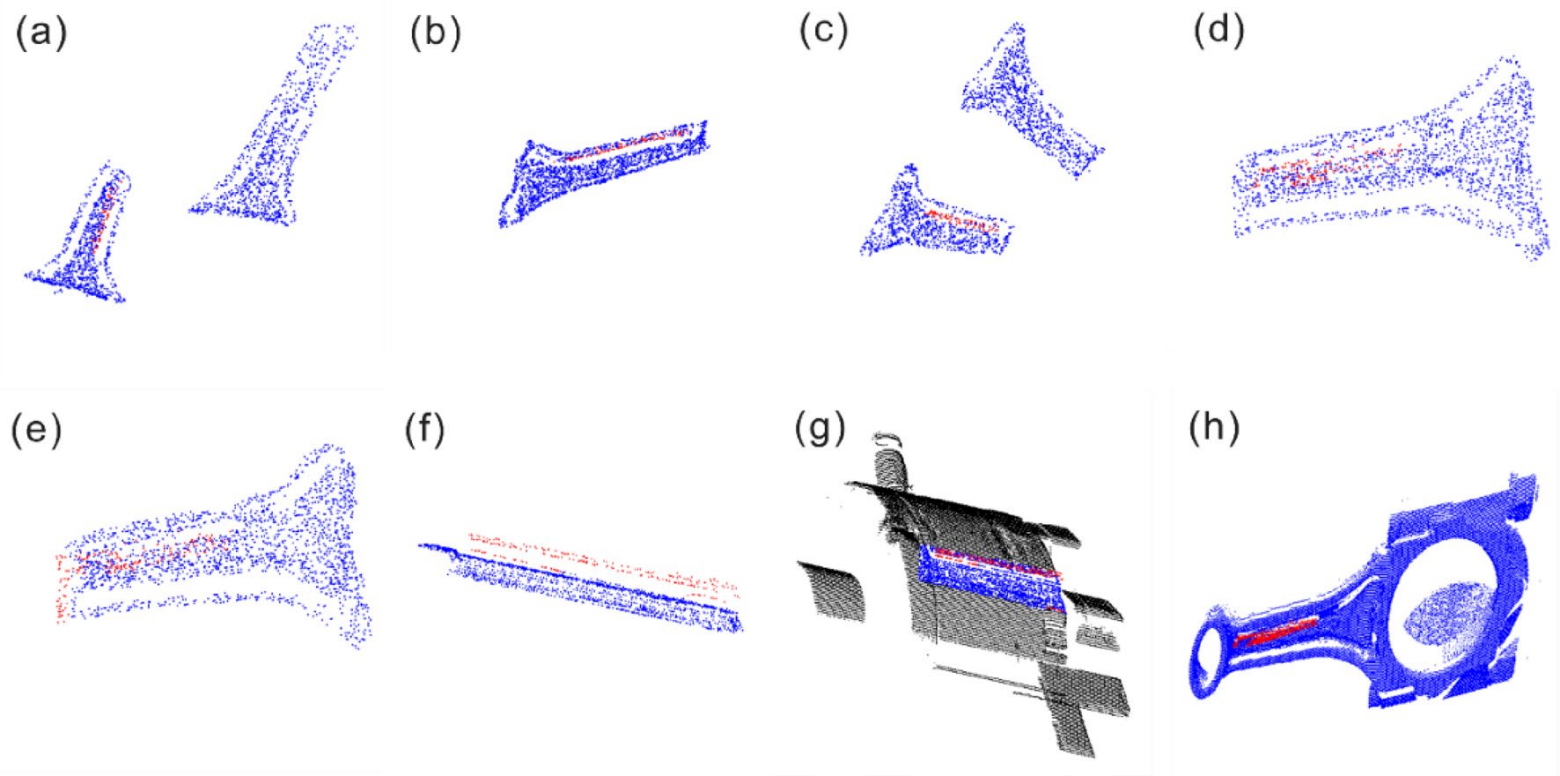
Partial dataset illustrations: (**a**–**d**) represent a subset of the sample dataset; (**e**,**f**) represent a subset of the validation dataset; (**g**,**h**) represent a subset of the test dataset.

Due to the current absence of point cloud datasets specifically tailored for line-structured scanning with mixed-reflection outlier points, the point cloud datasets used in this study were all custom-created. These datasets mainly consist of 3D point cloud models of the WD-2 connecting rod workpiece and local point clouds from the robotic arm fixture, captured from different scanning poses. The complete 3D point cloud model contains approximately 80,000 points. However, the number of outlier points in the reflective areas was insufficient. Therefore, manual cropping was applied to retain the outlier points within the reflective regions and the surrounding points. Subsequently, the dataset was downsampled to 2048 points to create the dataset, and labels were assigned to distinguish between outlier points and surface points. A total of 32 real workpiece datasets were collected. To augment the dataset, the original point clouds were subjected to rotations, translations, and scaling operations, resulting in 1024 augmented data samples. The dataset is illustrated in Fig. 10.

The training dataset consists of both the sample dataset and the test dataset. The sample dataset comprises two original point cloud sets of different shapes: WD-2 point clouds with 896 pose point clouds and local point clouds of the robotic arm fixture with 128 pose point clouds. The test dataset includes 112 connecting rod point clouds and 16 local point clouds of the robotic arm fixture. The validation dataset is composed of 14 connecting rod workpieces and 2 robotic arm fixture point clouds, designed to validate the effectiveness of outlier point identification resulting from mixed reflections, as shown in Table 1.

**Table1:** Dataset composition.

Dataset	sample	Connecting rod workpiece	Mechanical arm fixture
Sample set	1024	896	128
Test set	128	112	16
Verification set	16	14	2

### Improved PointNet outlier recognition network

The model was trained using the self-made dataset on a Windows 10 64-bit Professional Edition operating system with an Intel(R) Core(TM) i5-9500 CPU @ 3.00 Hz processor, NVIDIA RTX 3060Ti GPU with 8 GB of VRAM, 16 GB of RAM, TensorFlow 1.3 framework, and Visual Studio Code 1.72 development platform.

The data augmentation was set to include random horizontal flipping and normalization. Other hyperparameters were configured as follows: batch size was set to 8, the number of input points was Batch Size * 1024, the maximum number of epochs was 250, the loss function was the multi-class cross-entropy calculation function, the optimizer selected was Adam, with an initial learning rate of 0.001. The learning rate was adjusted using the exponential decay method provided by TensorFlow, with a decay rate of 0.7.

**Figure 11:**
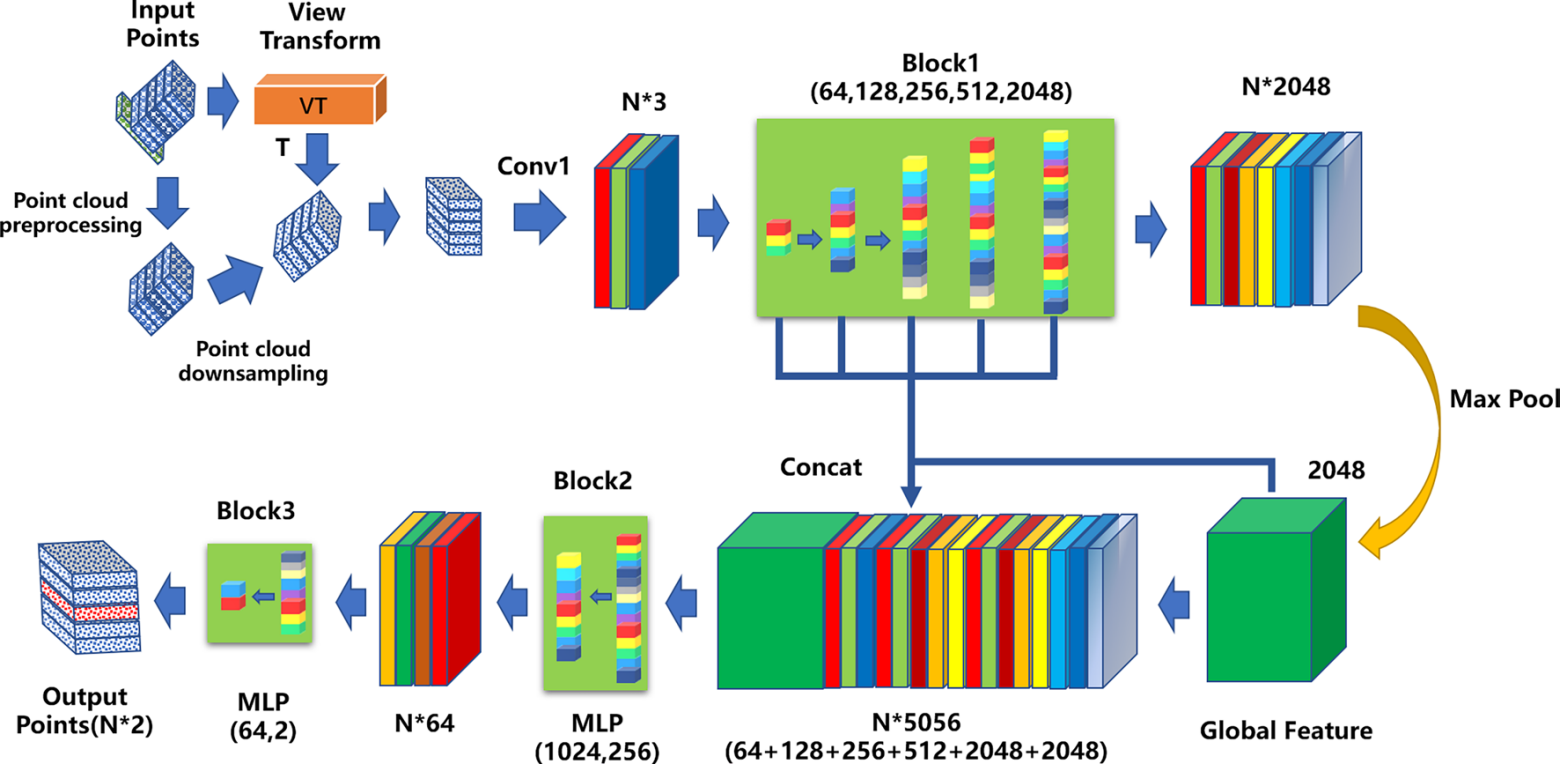
PointNet Outlier Recognition Network Based on View Transform.

Based on the specific viewpoint order of outliers generated by line-structured mixed reflection, this paper proposes an improved outlier recognition network based on PointNet, as shown in Fig. 9, by combining PointNet network. The input is the raw 3D point cloud, VT is the View-Transform point cloud view rotation, and the output is the normal point cloud and the outlier point cloud (Fig. 11).

The two types of point clouds generated as output are concatenated together to serve as the input for the subsequent network. Next, a convolution operation is applied to compress the three-dimensional coordinates (xyz) into one dimension with a single channel. Then, the compressed data is progressively expanded using multiple MLPs, resulting in channel sizes of [c1, c2, c3, c4, c5], while keeping a record of the channel sizes for [64, 128, 256, 512, 2048].

In this context, the MLPs are implemented using weight-sharing convolutional layers with kernel sizes of [1,1] to effectively capture local features in the point cloud while reducing computational complexity. The generated data is subjected to Max Pooling to extract global features. These global features are concatenated with the recorded data from different channel sizes to obtain Concat Features. The global features are automatically broadcasted to the appropriate dimensions, ensuring that the concatenated feature has a channel size of *c*_*out*_*.*The concatenated feature is then processed through multiple MLPs to obtain Point Features, and finally, a fully connected layer is designed based on the task requirements. In this paper, the input and output of the final fully connected layer are [64, 2], which is used to accomplish a binary classification task.The network structure for feature extraction is shown in Table 2.

**Table 2:** The network structure of feature extraction.

Block	Implementation	Kernels	Input	Channel	Activation function
Conv1	Conv	1*3	x,y,z	1	–
Block1	Conv*	1*1	Conv1	3, 64, 128, 256	ReLU
Block2	Conv*	1*1	Concat Features	512, 2048	ReLU
Concat1	Concat	–	Block1, Global Features	5056	–
Block3	FC	–	Point Features	2048	ReLU

Conv denotes the convolution operation, while Conv* signifies the convolution operation with weight sharing. Together, they form the MLP structure, with FC representing the fully connected layer.

In “PointNet Outlier Recognition Network Based on View Transform” Section of this paper, it is explained that the outlier points generated by the line structured system when capturing concave structures exhibit a particular orderliness from specific viewpoints. Therefore, the proposed View-Transform method is capable of rotating the point cloud model generated by this system to an orderly viewpoint. In PointNet, there exists a T-Net structure, which extracts features from the input point cloud to obtain a rotation matrix with the aim of aligning the input point cloud with a pose that exhibits stronger features. However, in the context of this system, the outlier points exhibit the strongest features from the orderly viewpoint. Additionally, with regards to the T-Net in PointNet, it performs relatively weakly in practice. In PointNet++, the authors have abandoned the use of T-Net.

Due to the uniqueness of the outlier points, it is beneficial to apply the optimal feature-based rotation to the point cloud during the input stage, replacing the T-Net. This allows the subsequent PointNet feature extraction network to capture stronger outlier point features, enhancing the network's ability to recognize outliers. Therefore, the View-Transform proposed in this paper is more akin to a preprocessing operation for the input point cloud of the point cloud network and does not alter the structure of the subsequent feature extraction network. Taking PointNet as an example, it is verified that View-Transform outperforms T-Net, and the optimal pose is beneficial for point cloud feature extraction. Furthermore, in Table 3, the introduction of View-Transform into the point cloud input preprocessing stage of PointNet++ is tested and similarly improves the accuracy of PointNet++ in recognizing outlier points.

**Table 3:** Experimental network parameters.

Net	Batch size	Learning rate	Activation function	Decay rate	mIoU (%)
PointNet	8	0.001	ReLU	0.5	45.369
View-Transform-PointNet	8	0.001	ReLU	0.5	48.260
PointNet++	8	0.001	ReLU	0.5	87.349
View-Transform -PointNet++	8	0.001	ReLU	0.5	95.216

Based on the experimental results, the View-Transform-PointNet proposed in this paper has shown an improvement of 2.891% in mIoU and 4.645% in mAcc compared to PointNet. Meanwhile, the View-Transform-PointNet++ has shown an improvement of 7.867% in mIoU and 2.777% in mAcc compared to PointNet++ . These results effectively demonstrate the significant improvement in point cloud recognition networks brought by the proposed View-Transform method.

**Figure 12:**
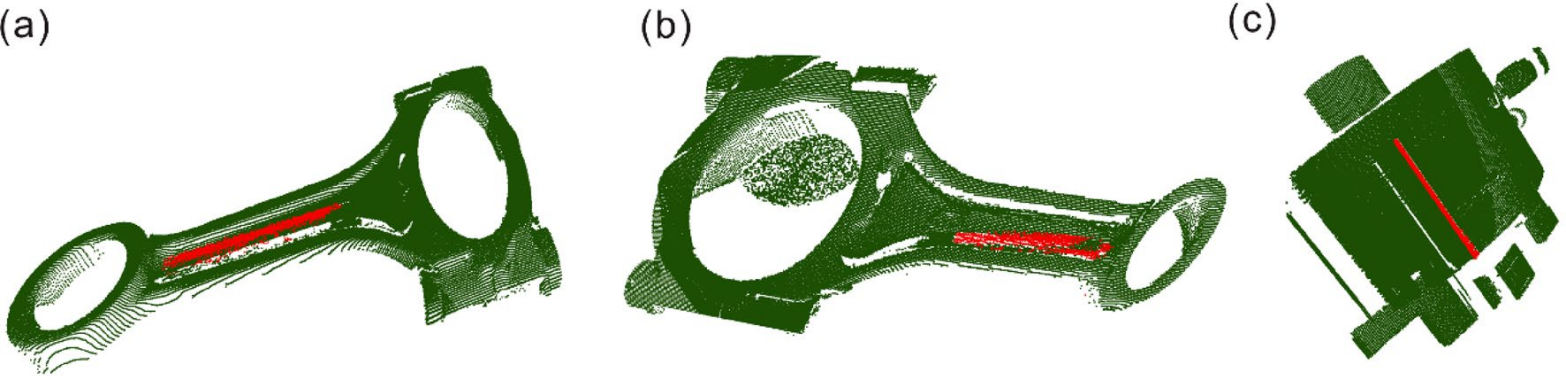
The segmentation effect diagram of the improved PointNet outlier recognition network (**a**) Link 1; (**b**) Connecting rod 2; (**c**) Robot arm fixture HFCY40.

The segmentation effect of the PointNet outlier recognition network improved based on View Transform is shown in Fig. 12.

### Experimental results and analysis of outlier location information restoration

In this paper, the automobile connecting rod workpiece is scanned by linear structured light; Then, preprocess and project the obtained point cloud model to measure the length of each outlier plane in the reflective area; Secondly, the gray value of structured light corresponding to the beginning and end positions of each outlier plane is collected in the image domain; Finally, based on the principle of optical hybrid reflection model and the relationship between triangulation structure, the distance between the ends of each outlier plane and the real surface is calculated. The parameters and processing results are shown in Table 4.

**Table 4:** Gray scale parameters of outlier plane structured light.

Outlier plane number	Euclidean distance of outlier plane ($d_{on}$)	Number of outlier plane point clouds (n)	Gray value atthe end ($I_{1}$)	Gray value at the beginning ($I_{2}$)	Gray scale variation coefficient (k)	Length to be repaired ($d_{l}$)
#1	2.19933428	23	128.266	174.469	1.742982057	6.03269228
#2	2.35317679	43	107.144	163.1111	1.641837794	6.21670669
#3	2.6302789	56	139.930	185.792	1.509048886	6.5994793
#4	3.3532646	100	143.380	193.917	1.20867879	7.4062801
#5	4.2865751	204	117.908	197.583	0.7206401	7.3756429
#6	5.1977345	302	148.579	233.094	0.259196592	6.5449622
#7	5.5687807	376	229.279	251.988	0.132634638	6.307398
#8	5.16374041	404	253.164	254.896	0.060046189	5.47372411
#9	3.8789068	332	255.000	255.000	0.000	3.8789068
#10	6.5326717	252	255.000	255.000	0.000	6.5326717
#11	7.1766251	158	255.000	255.000	0.000	7.1766251
#12	1.6292558	60	255.000	255.000	0.000	1.6292558
#13	1.1700644	38	130.832	193.533	0.980319293	2.317096318
#14	1.0003057	25	125.626	195.637	0.847909614	1.84847396
#15	1.0346494	28	102.207	175.811	1.075879028	2.147799445
#16	0.7900343	20	141.579	194.129	1.158344434	1.705161408
#17	0.8697882	19	82.506	174.615	0.872716021	1.628868137
#18	0.9281936	19	246.906	251.319	0.834126445	1.702470776
#19	0.7026071	12	213.490	232.846	1.14455466	1.506784868
#20	0.7051744	11	146.747	209.054	0.737413132	1.225179008
#21	0.6926074	14	250.642	252.656	1.163853029	1.498764587
#22	0.9089122	16	255.000	255.000	0.000	0.9089122
#23	0.5664053	15	245.749	249.656	1.367801382	1.3411909
#24	0.9868526	15	230.351	241.845	1.144510179	2.116327704
#25	0.6112723	17	253.468	253.893	2.604705882	2.201925842
#26	1.3423352	13	199.993	223.914	1.299527612	3.086734258
#27	1.2124161	15	149.227	197.056	1.211482573	2.681226739
#28	0.6933827	5	118.774	203.967	0.599028089	1.108739622

**Figure 13:**
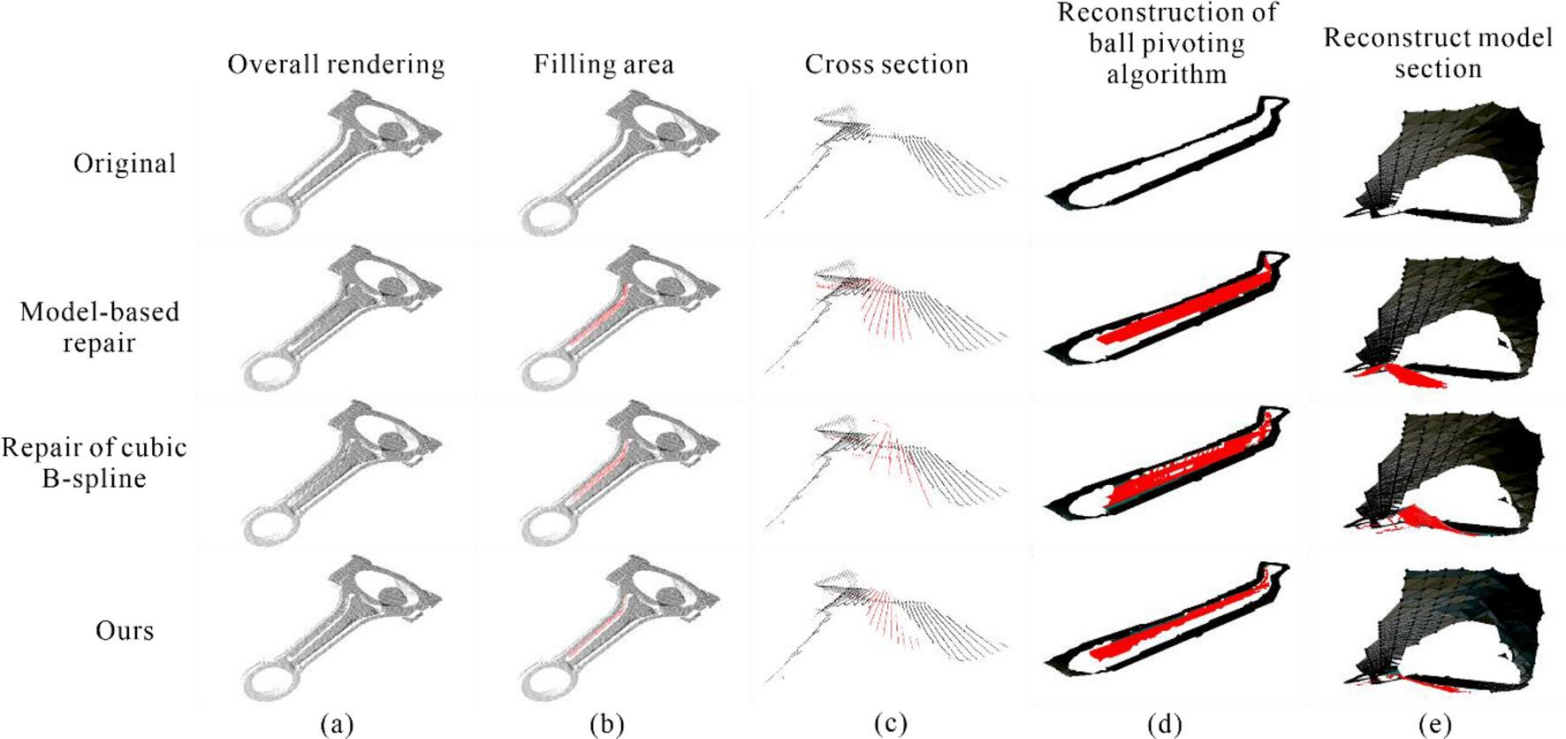
Schematic diagram for comparing the effects of various methods (**a**) Overall view; (**b**) Fill in the area; (**c**) Cross section; (**d**) Ball pivoting 3D reconstruction; (**e**) Rebuilding Model Sections.

After comparing our proposed method with neighborhood feature patching and cubic B-spline interpolation patching algorithms, the Ball Pivoting algorithm was used for 3D surface reconstruction of the holes and fill areas, with a clustering radius parameter set to 20 and an angle threshold parameter set to 90. The results are shown in Fig. 13.

From Fig. 13a,b, it can be seen that each methodology is able to fill the hole re-gion effectively in the whole. Our method is based on outlier planes for point cloud hole repair, and because of the limitation of the sampling frequency of the hardware device, so that the number of outlier planes is limited, the number of point clouds filled with hole point clouds is lower than the model-based repair and the repair of cubic B-spline inter-polation.

From the schematic diagram of the cross section in Fig. 13c, the model-based re-pair has got the extraction, migration, and then filling of the hole region for the features around the hole. The generated point cloud has weak fluctuations in the overall filling of the hole region, and more consistent with the point cloud features at the right edge of the hole, closer to the left edge of the hole, greater discrepancy between the point cloud and the real surface traits. The method of repair of cubic B-spline interpolation, which fills the missing point cloud by fitting the hole boundary at the cross-section, the curve fitting effect fluctuates more when more point clouds are missing in the middle of the point cloud hole, generating drastic changes in the point cloud, with obvious differences in the left side of the region with large fluctuations. The method in this paper has a strong consistency be-tween the global features and the point cloud features at the edge of the hole, and there is less variability with the actual surface traits in the area with greater curvature on the left side of the hole.

Overall, all methods can effectively fill the hole areas as shown in Fig. 13a,b. In this paper, our method repairs the point cloud holes using outlier planes. Due to the limited number of outlier planes resulting from hardware sampling frequency limitations, the number of point clouds for hole filling in our method is lower than that in the neighborhood feature and 3rd-order B-spline interpolation patching methods, as shown in Table 5.

**Table 5:** Various indicators of different algorithms in the hole area.

Object	Number of point clouds	Number of triangular mesh faces	Surface Area	Standard deviation between point clouds	CD
Original	6580	5523	479.208499	–	–
amodel-based repair	11,123	10,912	874.127821	0.915074	0.660563
bRepair of cubic B-spline interpolation	10,385	8806	893.506088	0.990167	0.668813
Ours	9172	8021	704.191745	0.828718	0.4471009

^a^The method of filling holes by model local similarity.

^b^Approximate interpolation method based on cubic B-spline curves.

**Figure 14:**
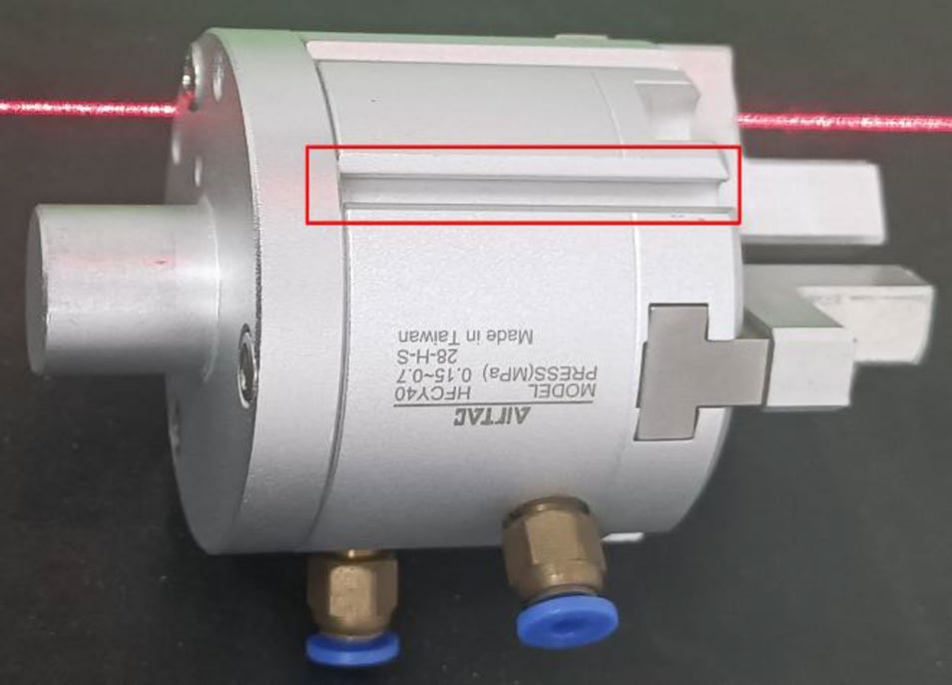
Reflective area of robot arm clamp.

**Figure 15:**
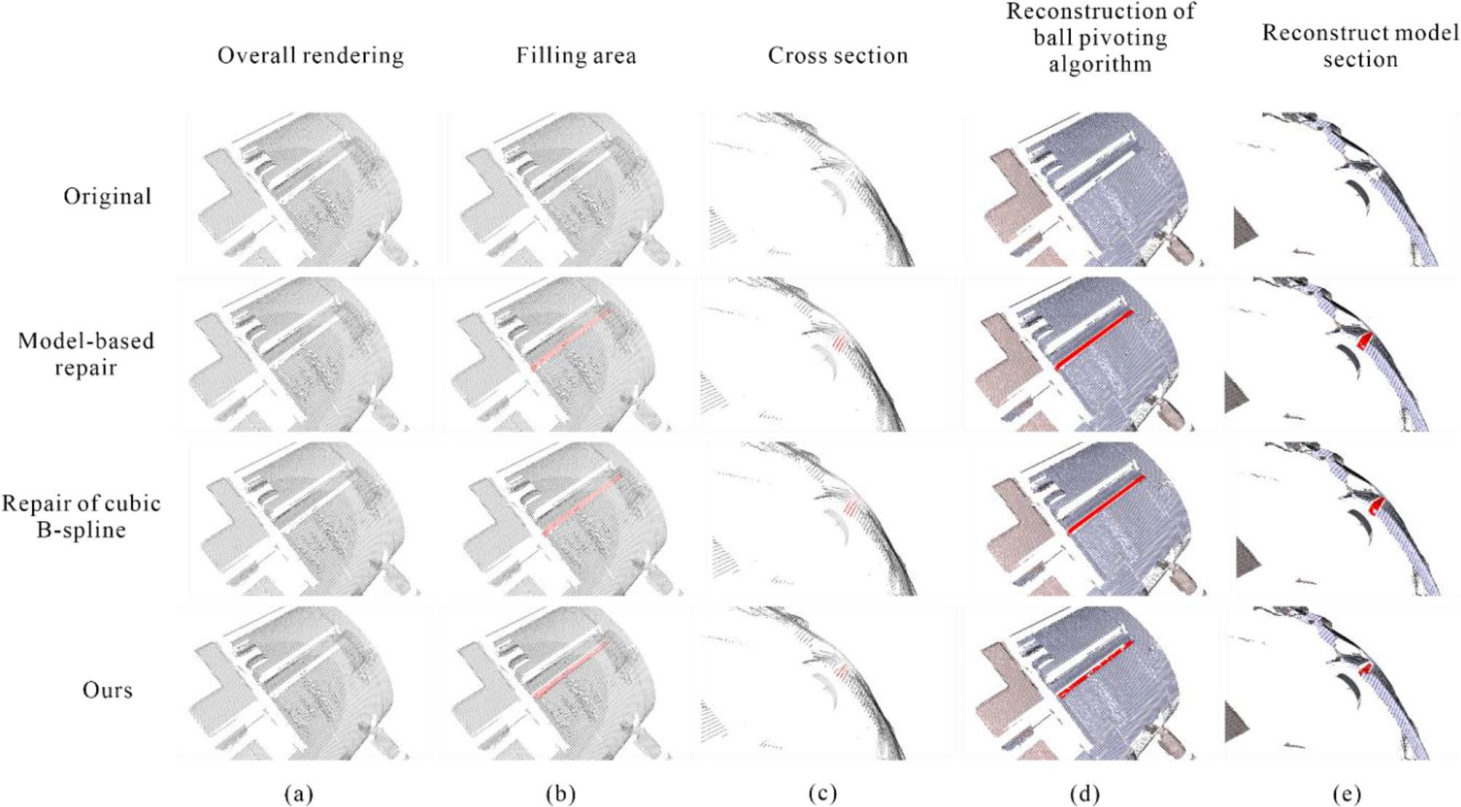
Diagram of effect comparison of each method.

Regarding the gripper of the robotic arm (Fig. 14), the hole filling effect of its point cloud is shown in Fig. 15. Under the camera view, the three methods perform similarly, as shown in Fig. 15d. However, in the cross-sectional view, only our proposed method is able to calculate the depth information based on the outlier points, while the hole is re-paired based on the surrounding point cloud using the model-based and cubic B-spline interpolation methods, which cannot restore the true surface shape at that point, as shown in Fig. 15e.

Overall, for connecting rod components, our proposed method exhibits superior hole-filling performance compared to model-based patching and cubic B-spline interpolation methods. Specifically, our method achieves a 39.4% increase in point cloud filling quantity, a 45.2% increase in triangle mesh surface quantity, and a 46.9% increase in filling area. Moreover, it results in smoother transitions between hole regions and surrounding point clouds, and effectively handles areas with high curvature while producing smaller errors. For robotic arm fixtures, our proposed method is capable of accurately detecting the shape of the real surface. However, limitations include lower filling quantity and filling area compared to model-based patching and cubic B-spline interpolation methods, due to hardware equipment sampling resolution and the restriction of the number of outlier planes.

### Experimental results and analysis of poisson surface reconstruction

**Figure 16:**
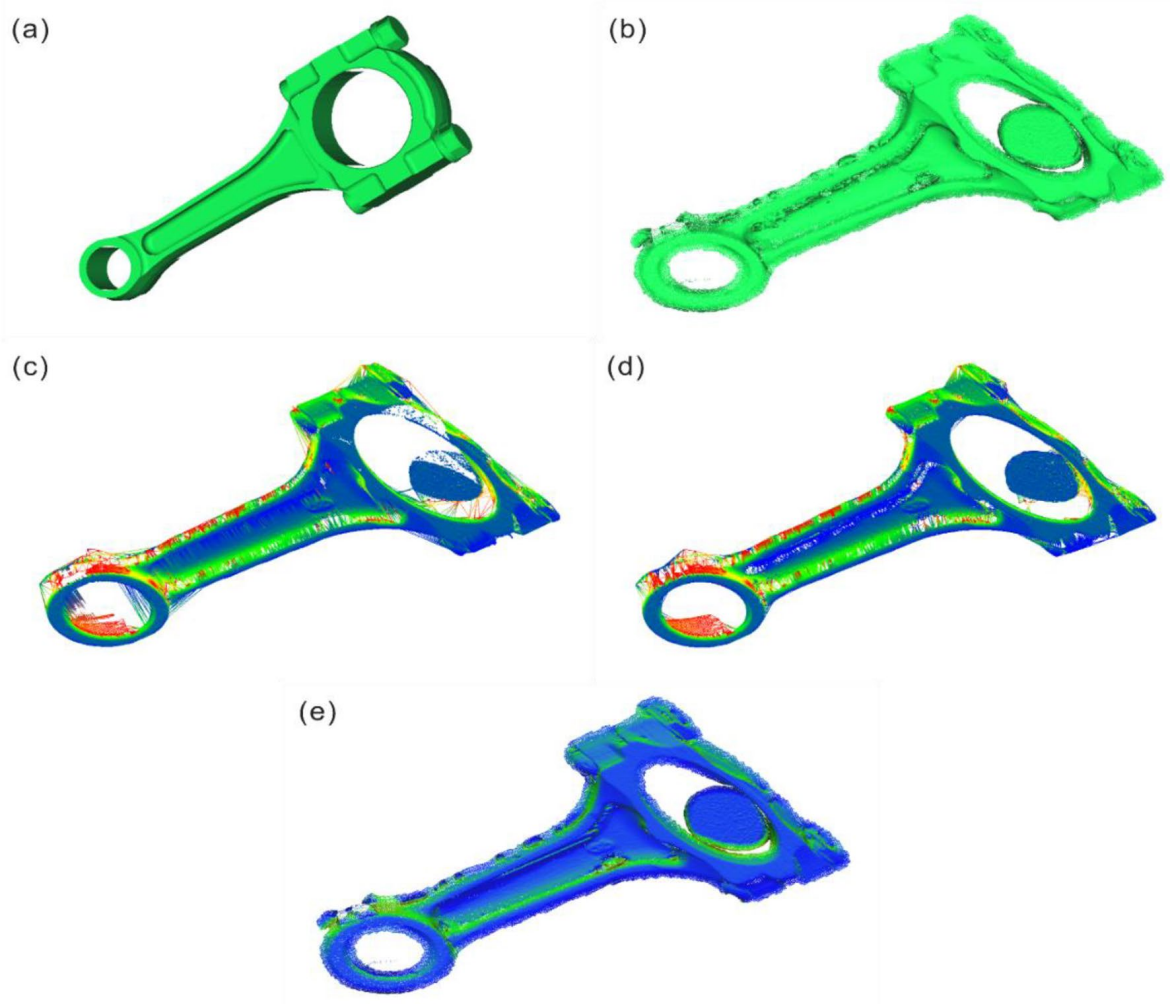
Standard connecting rod model, structured light scanning source model, Delaunay triangulation reconstruction and model comparison after repair (**a**) 3D model of standard connecting rod; (**b**) Initial model of line structure scanning; (**c**) Delaunay (XY plane) 3D reconstruction model; (**d**) Delaunay (Best fitting plane) 3D reconstruction model; (**e**) Poisson 3D Reconstruction Model.

Perform Poisson surface reconstruction on the repaired point cloud model mentioned above to obtain a complete 3D model, with the reconstruction effect shown in Fig. 16.

The comparison of the repair effect on the region is shown in Fig. 16.

**Figure 17:**
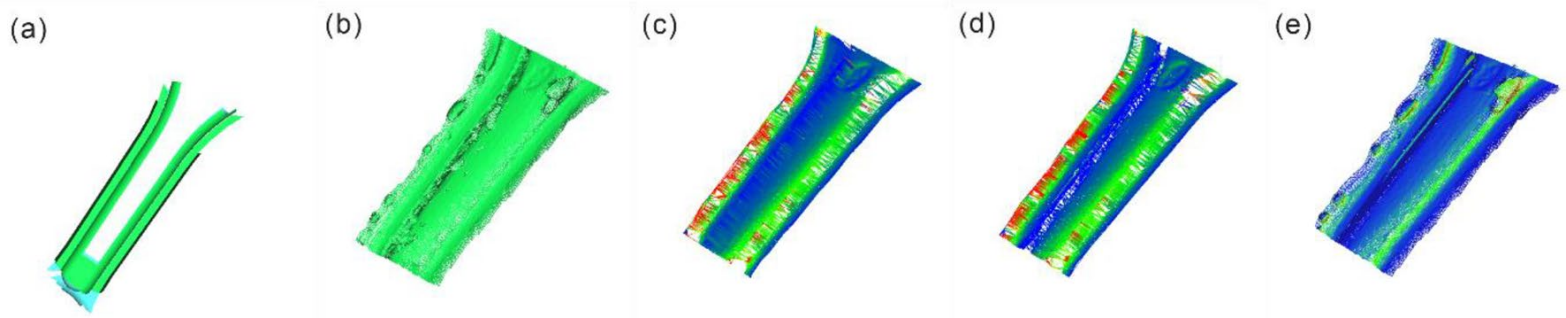
Comparison of repair area effects (**a**) Standard connecting rod 3D model; (**b**) Initial model of line structure scanning; (**c**) Delaunay (XY plane) 3D reconstruction model; (**d**) Delaunay (Best fitting plane) 3D reconstruction model; (**e**) Poisson 3D Reconstruction Model.

Based on the results of the repaired area, Delaunay 3D reconstruction performs better in the uniform distribution area of point cloud density, but its performance is poorer in the sparsely distributed point cloud density areas, such as the center of the hole and the edge of the model, as shown in Fig. 17c,d. On the other hand, the Poisson reconstruction method proposed in this paper partially restores information in the center of the hole, resulting in a more uniform distribution of point cloud density in this area compared to the unrepaired surface model. In terms of repair effect, the smoothness is better in the transition area of the concave surface edge, and the direction of the normal vector is roughly consistent in this area, as shown in Fig. 17e. However, the drawback is that there is still a gap between the repaired edge area and the standard model.

## Conclusion

Aiming at the problem of 3D reconstruction of highly reflective components, this paper proposes a PointNet neural network based on View Transform. By improving the alignment network in PointNet, it can automatically identify and extract outliers on the highly reflective surface scanned by line structured light; A repair method is proposed for the generated holes in the point cloud. The depth of the outliers is repaired through the distribution of outliers and structured light gray distribution, and the holes in the point cloud are filled. Finally, by performing Poisson surface reconstruction on the repaired hole area point cloud and global point cloud, the three-dimensional surface reconstruction of strongly reflective components is achieved. Compared with PointNet, the View Transform PointNet proposed in this article has an increase of 2.891% in mIoU and 4.645% in mAcc; Compared with PointNet++ , View Transform PointNet++ has increased mIoU by 7.867% and mAcc by 2.777%. Effectively proving that the View Transform proposed in this article has a significant improvement effect on point cloud recognition networks. This article explores a method for repairing point cloud depth information by filling reflective area point cloud holes with outlier point clouds to obtain high-precision component point cloud models. Compared with the unrepaired, the number of point cloud fills increased by 39.4%, the number of triangular mesh faces increased by 45.2%, the surface area increased by 46.9%, and the point cloud chamfer distance was 0.4471009; Compared with existing geometric restoration methods, it has the advantages of smaller standard deviation and stronger smoothness, and is more in line with the true surface characteristics of objects. The method proposed in this article for repairing point cloud holes based on outliers has been validated, which can effectively repair point cloud holes and improve model accuracy. Compared with the Delaunay surface reconstruction method, the number of triangular meshes increases by 97.9%; Compared with the original model registration error, the average distance is increased by 5.26%, and the standard distance is increased by 3.43%; Compared with the unrepaired model, the calculation time is reduced by 256 ms; In terms of repair effect, reduce errors in boundary areas, improve smoothness, and reduce sharp edges. Verify that the Poisson surface reconstruction used in this article can effectively achieve the reconstruction of point cloud models and effectively compensate for the shortcomings of repair methods.

There is still great room for improvement in identifying outliers and repairing point cloud holes in this article. The specific content of future work is as follows:

1. PointNet outlier recognition network based on View Transform. In terms of data set, due to the existing public data set, the point cloud data set that lacks line structured light scanning and still retains reflective outliers, there are fewer samples, less data and other defects in the point cloud data set; In addition, due to the reflective outliers generated by line structured light scanning, which have the characteristics of ordered specific viewing angles, the traditional point cloud enhancement methods are not applicable, and can not generate data set samples indirectly. Therefore, the improvement direction in data set: (1) In terms of online structured light scanning, multiple workpieces should be scanned to increase the number and type of samples; (2) In the aspect of point cloud enhancement of data set, starting from the characteristic of outliers generated by line structured light scanning, a point cloud data enhancement method based on this characteristic is proposed to expand the point cloud data set.
2. A point cloud hole repair method based on mixed reflection outliers. Due to the special nature of the repair method in this article, the area of the filled area depends on the number of outliers generated, and can only fill the area where mixed reflection outliers are generated; In addition, in terms of point cloud repair effect, the repair effect of the edge area of the concave structure still needs to be improved to make its transition smoother. Therefore, in response to the region limitation issue of point cloud repair methods, improvement directions are proposed: (1) in response to the characteristics of outliers generated by line structure scanning, point clouds are generated in areas without reflection; (2) Based on the point cloud of the repaired hole area, the point cloud of the repaired area is migrated to the unfilled area using a similar feature transfer method; (3) By using point cloud deep learning methods, feature extraction is performed on point clouds in repaired areas, and point clouds are generated in unfilled areas.

## Data Availability

Data underlying the results presented in this paper are not publicly available at this time but may be obtained from the authors upon reasonable request. Please contact Miao Longxin.(evange563@foxmail.com).
